# The Expression and Survival Significance of FOXD1 in Lung Squamous Cell Carcinoma: A Meta-Analysis, Immunohistochemistry Validation, and Bioinformatics Analysis

**DOI:** 10.1155/2022/7798654

**Published:** 2022-05-14

**Authors:** Fang Xie, Yunhui Li, Bin Liang

**Affiliations:** ^1^Medical Basic Experimental Teaching Center, China Medical University, Shenyang 110122, China; ^2^Clinical Laboratory, PLA North Military Command Region General Hospital, Shenyang 110003, China; ^3^Bioinformatics of Department, Key Laboratory of Cell Biology, Ministry of Public Health, And Key Laboratory of Medical Cell Biology, Ministry of Education, School of Life Sciences, China Medical University, Shenyang 110122, China

## Abstract

Accumulating evidence demonstrated that FOXD1 dysregulation was correlated with a broad spectrum of malignancies. However, litter is known about the role of FOXD1 in the progression of lung squamous cell carcinoma (LUSC). We conducted the comprehensive bioinformatics analysis to investigate FOXD1 expression in LUSC from TCGA and GEO datasets, and validated the FOXD1 expression pattern in clinical samples using immunohistochemistry method. ESTIMATE and CIBERSORT algorithms were performed to assess the relationship of FOXD1 and tumor microenvironment and immune cell infiltration. Our study showed that FOXD1 expression was significantly upregulated in LUSC tissues in TCGA dataset, validated by GEO datasets and clinical samples. In TCGA dataset, Kaplan-Meier curves showed that high FOXD1 expression was significantly correlated with favorable prognosis in LUSC patients. Moreover, FOXD1 expression has an impact on immune score and the proportions of immune cell infiltration subgroups. Finally, we predicted FOXD1 may be involved in many immune-related biological functions and cancer-related signaling pathways. Taken together, FOXD1 was upregulated in LUSC tissues, and FOXD1 expression could be a potential prognostic marker. FOXD1 might be associated with tumor microenvironment and perhaps a potential target in the tumor immunotherapy.

## 1. Introduction

According to the last GLOBOCAN estimates, the incidence and mortality of lung cancer ranked first worldwide in 2018, accounting for 2,093,876 new cases (11.6% of all new cases of malignant tumors) and 1,761,007 deaths (18.4% of all tumor deaths) [1]. Lung cancers are divided into two main subtypes, non-small cell lung cancer (NSCLC) and small cell lung cancer (SCLC). Lung squamous cell carcinoma (LUSC), a subtype of NSCLC, represents of 30% of NSCLC [2]. Previous studies demonstrated that the molecular mechanism of LUSC is associated with gene mutation, multiple altered expression of genes and pathways, and chromosomal instabilities in the progression of LUSC [3]. Moreover, smoking is one of the major risk factors in the development and progression of LUSC, and the rate of smoking exposure in LUSC patients exceeds 90% [4]. Although diagnosis and treatment have made remarkable progress in recent years, no specific biomarkers or relatively optimal targeted therapies have been identified for LUSC patients [5]. In LUSC patients, the 5-year overall survival rate for stage I and stage II patients is approximately 40%, while the overall survival (OS) rate is less than 5% for stage III and stage IV patients [6]. Therefore, it is of importance to unveil the molecular mechanism of LUSC and explore the target therapy strategies for LUSC patients.

The forkhead box (Fox) transcription factors (TFs) play important roles in biological processes, including cell growth, differentiation, proliferation, apoptosis, and longevity [7, 8]. Accumulating evidence has demonstrated that their mutation and dysregulation have been correlated to a broad spectrum of malignancies [9]. FOXD1, as a new member of the FOX family, is mainly located on 5q13.2 and encodes a DNA-binding protein containing 465 amino acids. Recently, many studies indicated that FOXD1 was involved in the development and progression of different types of human cancers, and its dysregulation was mainly associated with cell proliferation, migration, invasion, radioresistance, and epithelial-to-mesenchymal transition (EMT) [10–12]. Sohei Nakayama, et al. demonstrated that knockdown of FOXD1 could suppress cell growth in lung cancer cell lines, and high FOXD1 mRNA level was correlated with poor prognosis [13]. Li D, et al. indicated that FOXD1 has oncogenic characteristics by activating vimentin in NSCLC cell lines [14]. In LUSC, miR-30a-5p may inhibit the proliferation and migration of LUSC cells by inhibiting FOXD1 expression [15]. Although FOXD1 is involved in the pathological development of lung cancer, especially in LUSC, the related functions of FOXD1 contributing to LUSC progress have remained largely unknown.

In the present study, we analyzed FOXD1 expression in LUSC using The Cancer Genome Atlas (TCGA) and Gene Expression Omnibus (GEO) datasets, and validated the result by immunohistochemistry method. Then, we evaluated the association of FOXD1 expression and clinical parameters. Subsequently, we analyzed the relation between FOXD1 expression and tumor microenvironment (TME), as well as the immune cell infiltration.

## 2. Materials and Methods

### 2.1. Data Collection

**Figure 1:**
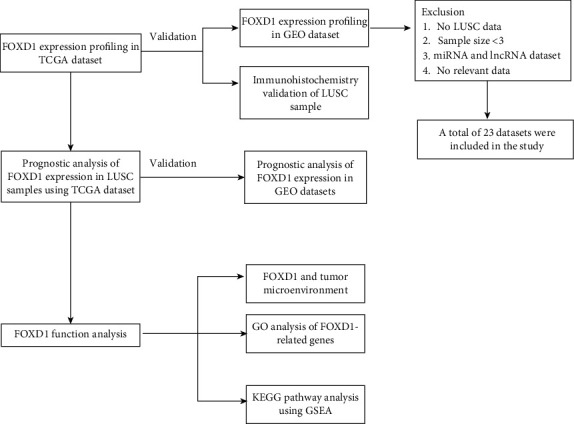
Schematic diagram of this study.

The transcriptome data and clinical information of LUSC were downloaded from TCGA database (https://www.cancer.gov/tcga). A total of 495 LUSC samples with detailed clinical information were enrolled in the study, as shown in Table 1. We searched GEO database (http://www.ncbi.nlm.nih.gov/geo/) to collect LUSC datasets up to December, 2020.The search terms included the following keywords: (lung) AND (tumor OR cancer OR carcinoma OR neoplasm). Each GEO dataset should meet the following criteria: (1) The sample includes cancer tissue and normal tissue; (2) The number of each group was greater than three. FOXD1 expression data was extracted from TCGA and GEO datasets. Then, the flow diagram of the study process was shown in Figure 1.

### 2.2. FOXD1 Expression in LUSC Samples from TCGA and GEO Datasets

FOXD1 expression was compared between LUSC tissues and adjacent noncancerous tissues in TCGA dataset. Subsequently, a total of 23 GEO datasets were included in the meta-analysis. The features of GEO datasets were listed in Table 2. We analyzed FOXD1 expression in all GEO datasets using Review Manager software (RevMan). The standard mean difference (SMD) and 95% confidence interval were used to estimate the expression pattern of FOXD1. Evidence of bias was assessed by visual funnel plots.

### 2.3. Immunohistochemistry Validation

Primary LUSC tissues were collected from LUSC patients, who underwent surgical resection in Shengjing Hospital, China Medical University. The study was approved by the Ethics Committee of Shengjing Hospital, China Medical University. All studies involving human participants were in accordance with the Declaration of Helsinki. All enrolled participants provided written informed consent.

First, we sectioned paraffin-embedded tissue blocks into 4-*μ*m thick sections for immunohistochemistry (IHC). All samples were deparaffinized and rehydrated in a series of xylene and graded ethanol solutions. The citrate buffer (pH 6.0) and 3% hydrogen peroxide were used for antigen retrieval and endogenous peroxidase activity blocking, respectively. Then, all slides were incubated with goat FOXD1 antibody (1 : 100, Abcam Company, #ab129324) overnight at 4°C. Tissue sections were then incubated with rabbit anti-goat horseradish peroxidase (HRP)-conjugated secondary antibody at room temperature for 1 h. Finally, all slides were visualized using DAB Horseradish Peroxidase Color Development Kit (Maixin Co., Fuzhou, China).

### 2.4. Association of FOXD1 Expression and Clinical Parameters

The association of FOXD1 expression and clinical parameters were evaluated using TCGA dataset. The clinical parameters included age, gender, distant metastasis, clinical stage, tumor size, node metastasis, and smoking. *P* <0.05 was considered as statistically significant.

### 2.5. Prognosis of FOXD1 Expression in LUSC Patients

The prognosis analysis was conducted in TCGA (n =495 cases), GSE3141 (n =53 cases), GSE13213 (n =117 cases), GSE14814 (n =52 cases), GSE37745 (n =66 cases), and GSE50081 (n =43 cases) datasets. We divided LUSC patients into two groups according to mean of FOXD1 expression, high FOXD1 expression group and low FOXD1 expression group. Kaplan-meier curve was plotted to evaluate the prognostic value of FOXD1, examined by Log-rank test. In TCGA dataset, the univariate and multivariate Cox proportional hazards models were performed orderly to screen the prognostic factors among the clinical parameters, as well as FOXD1 expression. *P* <0.05 was considered as statistically significant. Then, a nomogram was constructed on the basis of FOXD1 expression and clinical parameters, which were the independent prognostic factors in multivariate Cox regression analysis. Moreover, we used C-index and calibration plots to evaluate the performance of the prediction model. The model was visualized using R programming (R 3.6.2; http://www.r-Project.org).

### 2.6. Correlation of FOXD1 Expression and Immune Cell Infiltration

The stromal cells and immune cells are two major components in TME. The levels of stromal cells and immune cells in LUSC tissues were computed by Estimation of Stromal and Immune cells in Malignant Tumor tissues using Expression data (ESTIMATE) tool (https://bioinformatics.mdanderson.org/estimate/) based on gene expression data. The stromal and immune scores were compared between high FOXD1 expression group and low FOXD1 expression group.

To further explore the influence of FOXD1 in the immune microenvironment, we evaluated the proportion of immune cell infiltration for each LUSC sample using the Cell-type Identification By Estimating Relative Subsets Of RNA Transcripts (CIBERSORT) algorithm. CIBERSORT is a deconvolution algorithm, which could infer the abundance of 22 immune cell types based on gene expression data [16]. Then, we compared the difference of immune cell infiltration between high FOXD1 expression group and low FOXD1 low expression group. Furthermore, we analyzed the correlation of FOXD1 expression and immune cell subtypes. A *P* <0.05 was considered as statistically significant.

### 2.7. Function Enrichment Analysis for FOXD1-Related Genes

FOXD1-related genes were screened using LinkedOmics online tool (http://www.linkedomics.org/login.php). The screening threshold was set as: |*r*| >0.4 and *P* <0.05. Gene ontology (GO), including biological process (BP), molecular function (MF), and cell component (CC) were used to depict the function of FOXD1-related genes using STRING online tool (http://www.string-db.org). Then, we analyzed the potential miRNAs and transcription factors, which could regulate FOXD1 expression.

### 2.8. GSEA Analysis

We performed gene set enrichment analysis (GSEA) to explore the potential altered signaling pathways between high FOXD1 expression group and low FOXD1 expression group. The significantly enriched gene sets were identified with a nominal *P* value <0.05 and the |enrichment score| >0.4.

### 2.9. Statistical Analysis

All statistical analyses were conducted using SPSS 22.0 (SPSS, IL, United States) and R. 3.6.2 (https://www.r-project.org/). The correlation between FOXD1expression and various clinical features was analyzed by *t*-test and analysis of variance (ANOVA). Survival analysis was estimated by Kaplan-Meier curve, and tested by Log-rank test. Cox proportional risk regression models was used to assess independent risk factors for overall survival (OS) of LUSC patients. A *P* <0.05 was considered statistically significant.

## 3. Results

### 3.1. FOXD1 Expression Pattern in LUSC Samples

**Figure 2:**
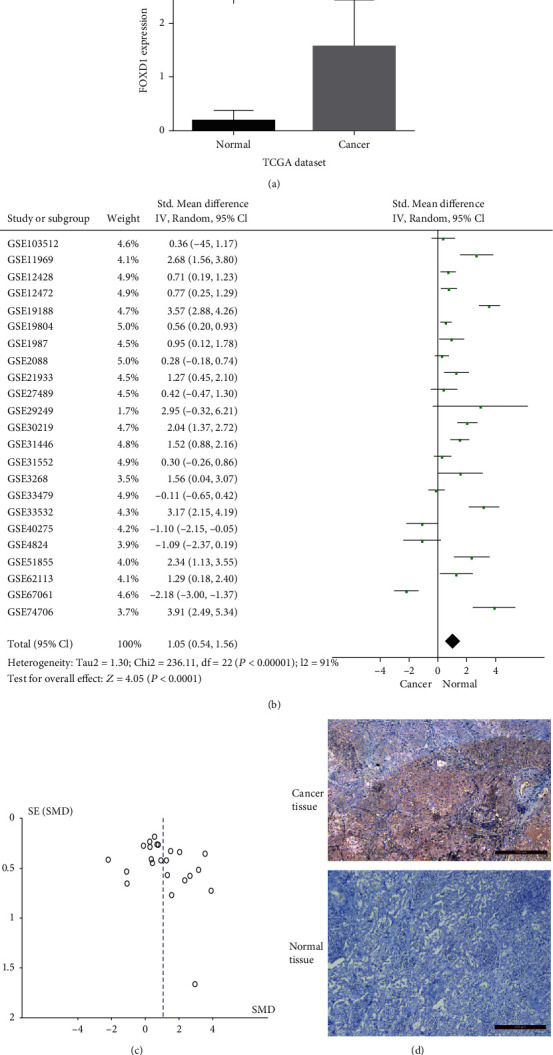
FOXD1 expression in LUSC samples. (a) FOXD1 expression in TCGA dataset (LUSC: 495 cases and normal control: 49 cases) . (b) FOXD1 expression in GEO datasets using meta-analysis (LUSC: 608 cases and normal control: 464 cases). (c) Funnel plot for the assessment of biases of the included datasets in meta-analysis. (d) Immunohistochemistry results of FOXD1 expression in LUSC tissues and normal tissues.

We first analyzed FOXD1 expression between LUSC tissues and adjacent noncancerous tissues in TCGA dataset, and found that FOXD1 was upregulated in LUSC tissues (*P* <0.05, Figure 2(a)). In order to verify the difference in FOXD1 expression in TCGA dataset, a comprehensive meta-analysis was conducted based on GEO datasets. A total of 23 GEO datasets were included in the study. The result showed that FOXD1 was upregulated in LUSC tissues in GEO datasets (SMD =1.05, 95% CI: 0.54-1.56, *P* <0.001, Figure 2(b)). The funnel plot of SMD for the included studies appeared to be symmetric, and displayed no publication bias (Figure 2(c)).

We also performed IHC to validate FOXD1 protein expression in clinical samples. IHC results indicated that FOXD1 in LUSC tissues was significantly upregulated than normal lung tissues (Figure 2(d)).

### 3.2. Association of FOXD1 Expression and Clinical Parameters

**Figure 3:**
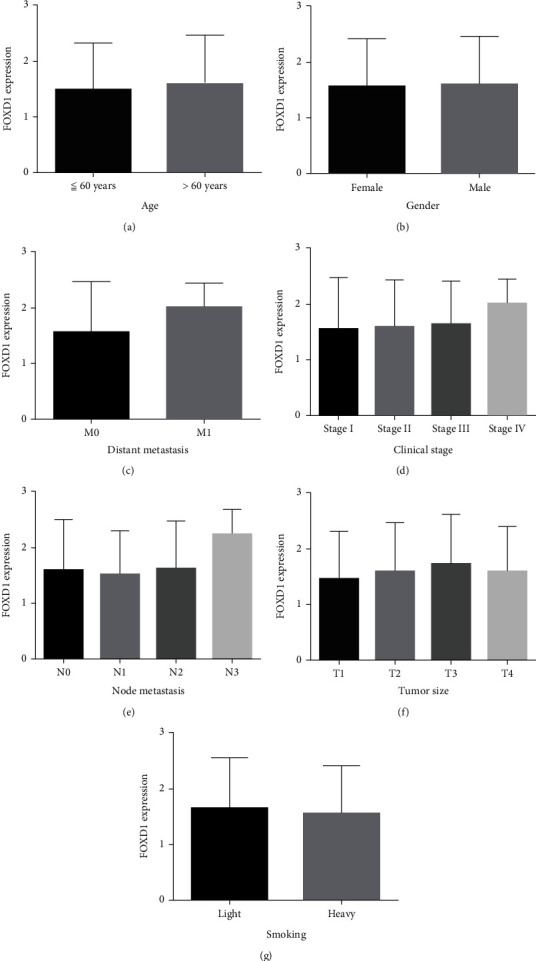
Association of FOXD1 expression and clinical parameters. (a) age (<=60 years/>60 years: 107 cases/382 cases); (b) gender (female/male: 129 cases/366 cases); (c) distant metastasis (M0/M1: 407 cases/7 cases); (d) clinical stage (I/II/III/IV: 242 cases/159 cases/83 cases/7 cases); (e) node metastasis (N0/N1/N2/N3: 316 cases/128 cases/40 cases/5 cases); (f) tumor size (T1/T2/T3/T4: 114 cases/228 cases/70 cases/23 cases); (g) smoking (light smoking/heavy smoking: 151 cases/332 cases).

Moreover, we analyzed the correlations between FOXD1 expression and clinical parameters, including age, gender, TNM stage, clinical stage, and smoking history. FOXD1 expression displayed no significant differences in all clinical characteristics (all *P* >0.05, Figures 3(a)–3(g)). In stage IV and N3 stages, FOXD1 showed a tendency of upregulation, but the difference was not significant (*P* >0.05).

### 3.3. FOXD1 Expression Was Positively Correlated with Overall Survival in LUSC Patients

**Figure 4:**
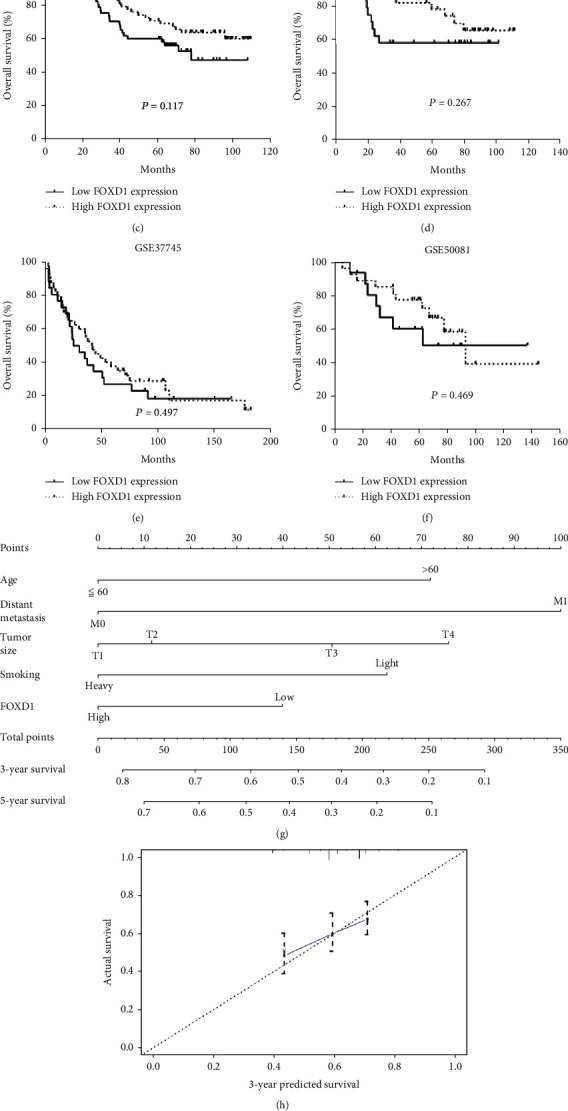
Correlation of FOXD1 expression and overall survival. (A-F) Survival plot of LUSC patients between high FOXD1 expression and low FOXD1 expression in TCGA, GSE3141, GSE13213, GSE14814, GSE37745, and GSE50081; (G) A nomogram integrating FOXD1 expression, age, distant metastasis, tumor size, and smoking for individual patients; (H) The calibration curves examining the predictive accuracy for 3-, and 5-year overall survival.

In TCGA dataset, we plotted Kaplan-meier curves, examined by Log-rank test, and found that high FOXD1 expression was positively correlated with OS in LUSC patients (*P* =0.036, Figure 4(a)). Moreover, we found that LUSC patients with FOXD1 expression had a longer OS in comparison with those with low FOXD1 expression in GSE3141, GSE14814, GSE37745, GSE13213, and GSE50081 datasets (Figures 4(b)–4(f)). Due to the small sample size, the differences were not significant.

As shown in Table 3, the univariate Cox regression analysis showed that FOXD1 expression (HR =0.750, 95% CI: 0.572-0.983, *P* =0.037), distant metastasis (HR =3.099, 95% CI =1.266-7.584, *P* =0.013), clinical stages (HR =1.557, 95% CI =1.131-2.144, *P* =0.007), tumor size (HR =1.651, 95% CI =1.195-2.281, *P* =0.002), and smoking history (HR =0.656, 95% CI =0.494-0.872, P =0.004) were associated with OS. Multivariate analysis indicated FOXD1 (HR =0.706, 95% CI =0.522-0.956, *P* =0.024) was an independent prognostic factor, as well as age (HR =1.866, 95% CI =1.205-2.891, *P* =0.005), distant metastasis (HR =2.512, 95% CI =1.021-6.179, *P* =0.045), tumor size (HR =1.516, 95% CI =1.042-2.205, *P* =0.030) and smoking history (HR =0.594, 95% CI =0.432-0.815, *P* =0.001).

### 3.4. Development of a Nomogram on the Basis of FOXD1 Expression and Clinical Factors

A nomogram integrating FOXD1 expression, distant metastasis, tumor size, and smoking history for OS prediction of LUSC patients was shown in Figure 4(g). In the nomogram, a high total point presents a worse clinical outcome. The C-index for the nomogram was 0.601 (95% CI: 0.822–0.861). The calibration plot of 3- and 5- years survival probability indicated a good consistency between the predictions by the nomogram and the actual observations (Figures 4(h) and 4(i)).

### 3.5. Relation of FOXD1 Expression and Tumor Microenvironment

**Figure 5:**
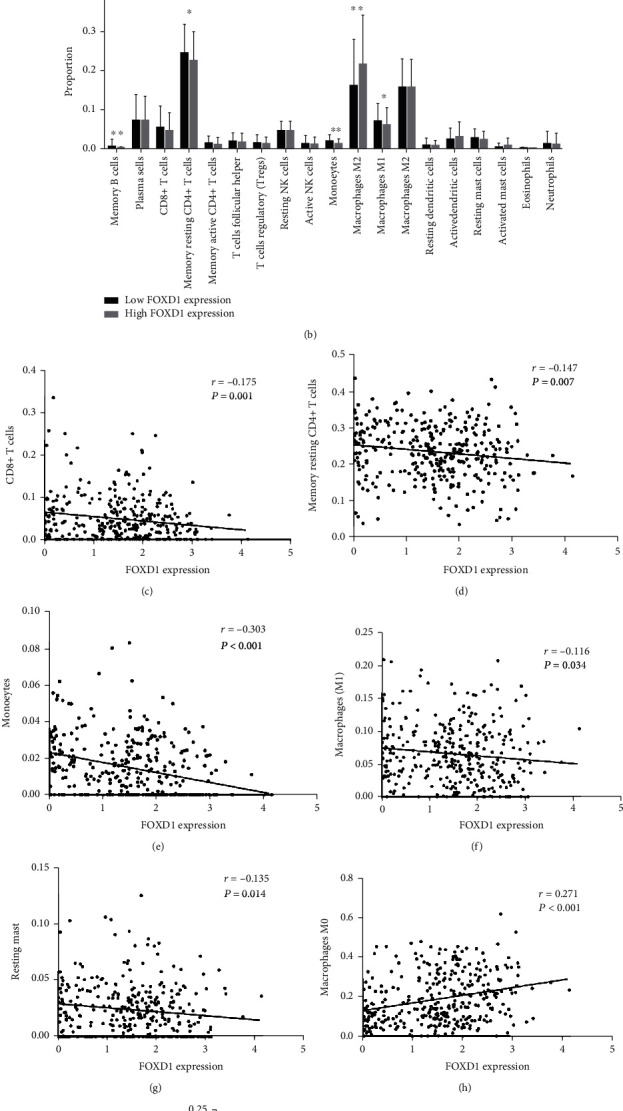
Correlation of FOXD1 expression and tumor microenvironment. (A) Differences of stromal score, immune score, and ESTIMATE score between high FOXD1 expression group and low FOXD1 expression group; (B) Profiling of tumor immune infiltrating cells between high FOXD1 expression group and low FOXD1 expression group; (C-I) Correlation of FOXD1 expression and immune cell subtypes, including CD4+ T cells, memory resting CD4+ T cells, monocytes, macrophages M1, resting mast, macrophages M0, and activated dendritic cells.

In the TCGA dataset, high FOXD1 expression group showed significantly lower stromal score (*P* <0.001) and immune score (*P* <0.001) in comparison with low FOXD1 expression group (Figure 5(a0). Moreover, the percentages of 22 immune infiltration cell subtypes were calculated using CIBERSORT algorithm. The proportions of memory B cell (*P* =0.002), resting memory CD4^+^ cells (*P* =0.012), monocytes (*P* <0.001), and M1 macrophages (*P* =0.030) in high FOXD1 expression group were significantly lower than low FOXD1 expression group (Figure 5(b)). Otherwise, the proportions of M0 macrophages (*P* <0.001) in high FOXD1 expression group were significantly higher than low FOXD1 expression group (Figure 5(b)). Furthermore, FOXD1 expression was negatively correlated with the proportion of CD8^+^ T cells (*r* = -0.175, *P* =0.001), resting memory CD4^+^ cells (*r* = -0.147, *P* =0.007), monocytes (*r* = -0.303, *P* <0.001), M1 macrophages (*r* = -0.116, *P* =0.034), resting mast (*r* = -0.135, *P* =0.014), respectively. FOXD1 expression was positively correlated with M0 macrophages (*r* =271, *P* <0.001) and activated DC (*r* =0.110, *P* =0.045), respectively (Figures 5(c)–5(i)).

### 3.6. Function Enrichment Analysis of FOXD1-Related Genes

**Figure 6:**
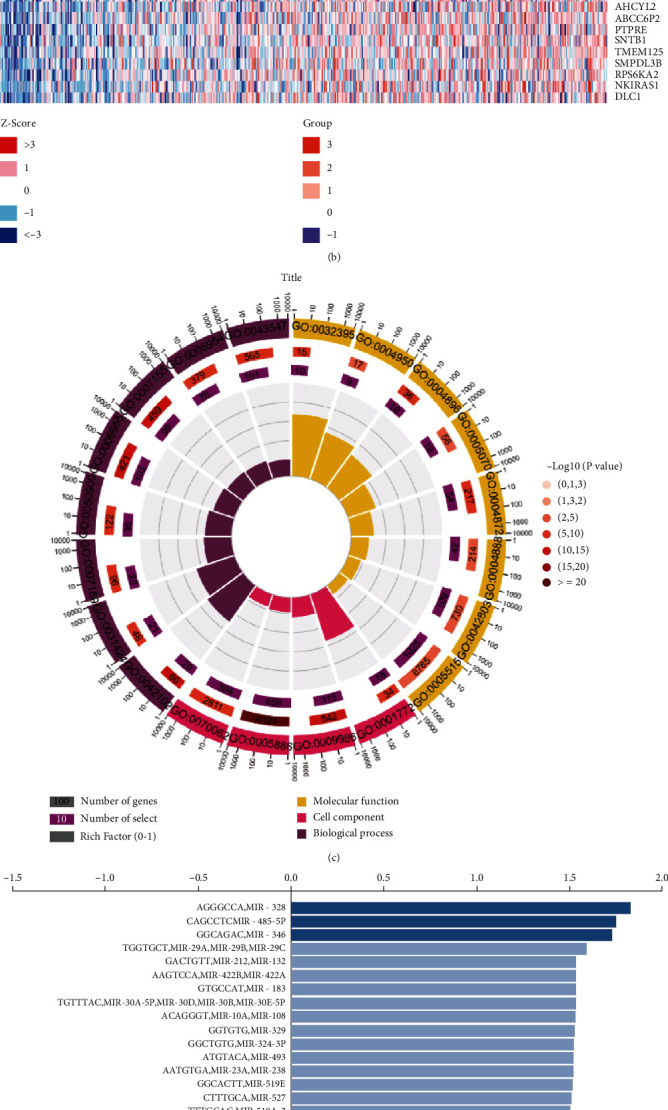
FOXD1-related genes and function enrichment analyses. (A) Positively correlated FOXD1-correlated genes; (B) Negatively FOXD1-correlated genes; (C) GO analyses of FOXD1-correlated genes; GO:0006955 immune response; GO:0042102 positive regulation of T cell proliferation; GO:0007155 cell adhesion; GO:0031424 keratinization; GO:0006954 inflammatory response; GO:0050900 leukocyte migration; GO:0007169 transmembrane receptor protein tyrosine kinase signaling pathway; GO:0043547 positive regulation of GTPase activity; GO:0005886 plasma membrane; GO:0009986 cell surface; GO: 0001772 immunological synapse; GO:0070062 extracellular exosome; GO:0004872 receptor activity; GO:0004896 cytokine receptor activity; GO:0032395 MHC class II receptor activity; GO:0005070 SH3/SH2 adaptor activity; GO:0004950 chemokine receptor activity; GO: 0004888 transmembrane signaling receptor activity; GO:0042803 protein homodimerization activity; GO:0005515 protein binding. (D) miRNAs prediction of FOXD1; (D) Transcription factors prediction of FOXD1.

A total of 360 FOXD1-related genes were identified through transcriptome co-expression analysis, including 308 positively correlated genes and 52 negatively correlated genes. We list the top 50 positive and negative FOXD1-related genes using heatmaps (Figure 6(a) and 6(b)). GO analyses showed that BP were mainly involved in immune response, positive regulation of T cell proliferation, cell adhesion, inflammatory response, and leukocyte migration; and MF were mainly involved in receptor activity, cytokine receptor activity, MHC class II receptor activity, SH3/SH2 adaptor activity, and chemokine receptor activity (Figure 6(c)). Moreover, we predicted the potential miRNAs (miR-328, miR-485-5P, and miR-346) and transcription factors (ZR5, VDR, NF-*κ*B, PEA3, ELF1, et al.), which could regulate FOXD1 expression (Figures 6(d) and 6(e)).

### 3.7. GSEA Analysis

**Figure 7:**
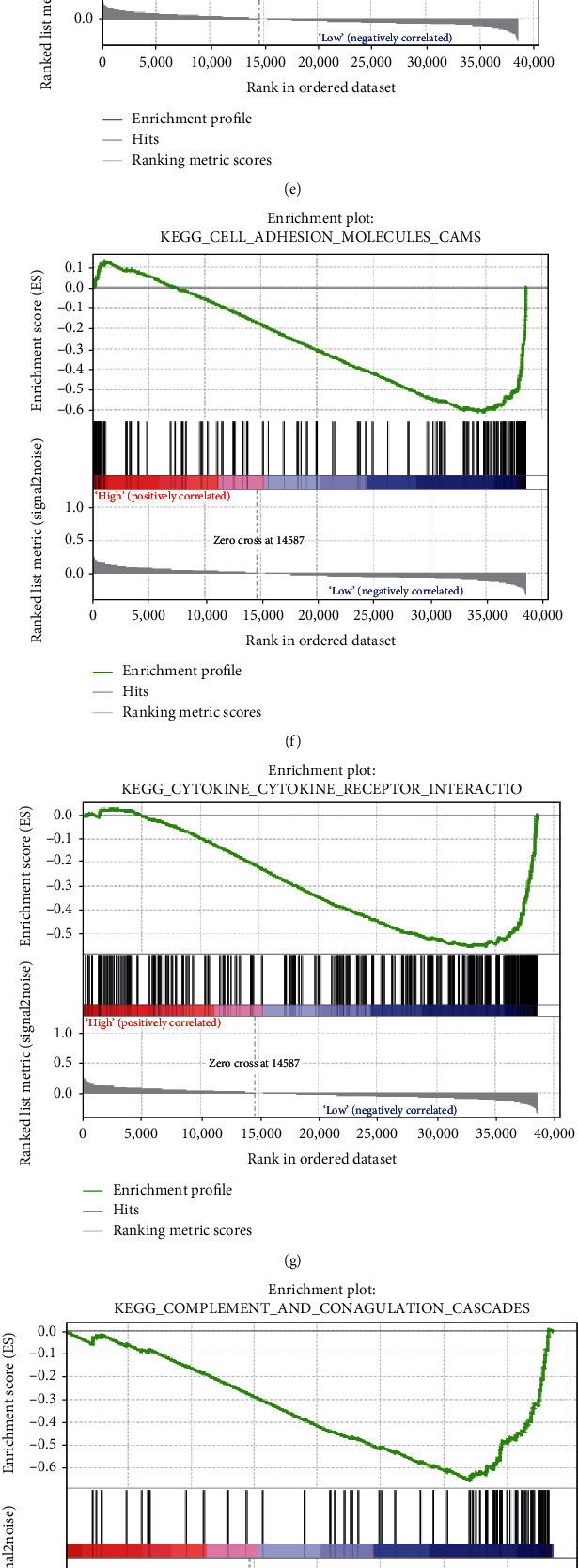
KEGG pathway enrichment analyses for high FOXD1 expression and low FOXD1 group. (A-D) GSEA results of high FOXD1 expression group; (E-L) GSEA result of low FOXD1 expression group.

The differentially expressed genes between high FOXD1 expression and low FOXD1 expression groups were conducted to perform KEGG pathway analyses. The results showed that enrichment pathways included glycosaminoglycan biosynthesis keratin sulfate, hedgehog signaling pathway, Wnt signaling pathway, and ERBB signaling pathway in high FOXD1 expression group (Figures 7(a)–7(d)). Otherwise, a series of immune-related pathways, such as antigen processing and presentation, cell adhesion molecules cams, cytokine cytokine receptor interaction, complement and coagulation cascades, natural killer cell mediated cytotoxicity, JAK-STAT signaling pathway, primary immunodeficiency, and chemokine signaling pathway, were mainly enriched in low FOXD1 expression group (Figures 7(e)–7(l)).

## 4. Discussion

FOX proteins are a superfamily of evolutionarily conserved transcription factors, which share ‘winged helix' DNA-binding domain (FOX domain) [9]. Recent studies identified that FOX domains have been found in over 100 proteins, ranging from FoxA to FoxQ subclasses [7, 17]. FOX family genes are involved in carcinogenesis as oncogenes and/or cancer suppressor genes. FOXD subfamily consists of 9 members, including FOXD1, FOXD2, FOXD3, FOXD4, FOXD5, FOXD6, FOXDL4, FOXDL5, and FOXDL6. Initially, the function of FOXD1 was identified in renal morphogenesis and the development of optic chiasm [18, 19]. Recently, the roles of FOXD1 have begun to be elucidated in the development and progression of tumors. In breast cancer, FOXD1 could induce G1 to S phase transition, thus promoting cell proliferation and chemoresistance [20]. In lung cancer, FOXD1 coupled with Gal-3 increased tumor growth and motility, whereas depletion of Gal-3 attenuated FOXD1-mediated tumorigenesis [21]. Moreover, FOXD1 promoted cell proliferation, migration and invasion in colorectal cancer cells by regulating the phosphorylation of ERK 1/2 pathway [22]. In melanoma, overexpression of FOXD1 enhanced drug resistance of melanoma cells [23]. Conversely, Woong Ju, et al. reported that FOXD1, as a transcription regulating gene, was significantly downregulated in chemoresistant epithelial ovarian cancer [24]. Therefore, FOXD1 plays important roles in many cancers, such as proliferation, metastasis, and drug resistance, but some cancers are on the contrary. In the present study, we found that the levels of FOXD1 mRNA were upregulated in LUSC tissues in TCGA and GEO datasets. Moreover, we collected clinical LUSC samples and validated FOXD1 protein upregulation using IHC experiments.

Among the clinical features, FOXD1 expression in our results was not significantly related to age, gender, TNM stage, and smoking history. However, FOXD1 expression displayed a decreasing tendency in advanced stage IV and N3 stages (Table 2). Unlike with our result, Sohei Nakayama, et al. reported that high FOXD1 mRNA level was significantly associated with squamous cell carcinoma, gender, and smoking history, and high FOXD1 expression predicted a shorter survival time than low FOXD1 expression [13]. Surprisingly, our survival analyses indicated that high FOXD1 expression was correlated with better clinical outcome using TCGA and GEO datasets. In Sohei Nakayama's study, FOXD1 expression was only significantly associated with squamous carcinoma, but not with other lung cancer subtypes. Moreover, Sohei Nakayama, *et al*. collected 90 lung cancer specimens, including squamous carcinoma, adenocarcinoma, neuroendocrine carcinoma and other lung cancer subtypes for prognosis analysis [13]. Maybe, due to sample diversity, our result was different from Sohei Nakayama's study. Furthermore, FOXD1 expression was upregulated in LUSC tissues, and high FOXD1 expression had longer survival time in our study. As every coin has two sides, one possible reason may be that FOXD1 was an inducer in the progression of LUSC, activating and/or inhibiting some potential signal pathways. Another explanation could be the existence of multiple influential factors that may lead to increased expression of FOXD1 under different circumstances. Other explanation was that the prognosis of LUSC was not determined by single factors, and the combination of FOXD1 and other influencing factors contributed to clinical outcomes in LUSC patients.

Regulation of the immune system is a critical part of anticancer therapies including immunotherapy, chemotherapy, and radiotherapy [25]. Our results indicated that high FOXD1 expression group showed a significantly lower immune score in comparison with low FOXD1 expression. Growing evidence has demonstrated that the immune response has antitumor effects, which immunologically mediated elimination of transformed cells has been widely accepted in the context of cancer for many decades [26]. FOXD1 low expression was companied by the low immune score, implying that FOXD1 was involved in the modulation of immune response in LUSC. Although still in debate, amounting evidence suggests that CD4^+^ T cells play an important role as a mediator in the maintenance and control of protective immune responses [27, 28]. Monocytes are one of the most abundant cells in the solid tumor bulk. Initially, monocytes contribute antitumor functions, and finally become tumor-supportive and immunosuppressive undergoing a phenotypic switch [29]. Macrophages are versatile immune cells, which are polarized to two opposite types, classically activated M1 macrophages and alternatively activated M2 macrophages. M1 macrophages, as an antitumor phenotype, exert an immune protective role by producing chemokines and cytokines to destroy tumor cells, whereas M2 macrophages protect cancer cells from antitumor immune responses and contribute to tumor progression [30, 31]. Our results showed that resting memory CD4^+^ cells, monocytes, and M1 macrophages in high FOXD1 expression group were significantly lower than low FOXD1 expression group, indicating that the immune protective function was dampened with FOXD1 downregulation.

The present study had some limitations. First, most samples were downloaded from TCGA and GEO datasets, and clinical samples in IHC experiments were absent from detailed clinical information, which may affect the results to some extent. Second, some important treatment information, such as chemotherapy and immunotherapy, were incomplete, so that we did not fully analyze the association of FOXD1 expression and immune therapy. In addition, functional experiments should be performed to further elucidate the molecular mechanisms of FOXD1.

## 5. Conclusion

In summary, our study illustrated that FOXD1 was upregulated in LUSC samples and could predict the prognostic outcome in LUSC patients. Moreover, FOXD1 expression was correlated with immune infiltration. Therefore, FOXD1 could be a new target gene, which provides a new therapeutic target in LUSC. Further studies are required to investigate its molecular function.

## Figures and Tables

**Table 1:** Clinical features in LUSC samples in TCGA dataset.

Clinical parameters	Samples	Percentage (%)
Age		
<=60 years	107	21.6
>60 years	382	77.2
NA	6	1.2
Gender		
Female	129	26.1
Male	366	73.9
Distant metastasis		
M0	407	82.2
M1	7	1.4
Mx	81	16.4
Clinical stage		
Stage I	242	48.9
Stage II	159	32.1
Stage III	83	16.8
Stage IV	7	1.4
NA	4	0.8
Tumor size		
T1	114	23.0
T2	288	58.2
T3	70	14.1
T4	23	4.7
Node metastasis		
N0	316	63.8
N1	128	25.9
N2	40	8.1
N3	5	1.0
NA	6	1.2
Smoking		
Light	151	30.5
Heavy	332	67.1
NA	12	2.4

NA: none available.

**Table 2:** The characteristics of included GEO datasets in meta-analysis.

Datasets	Contributor	Year	Country	Platform	Samples (cancer/normal)
GSE1987	Dehan E	2004	Israel	GPL91	20/9
GSE2088	Joans MH	2004	Japan	GPL962	48/30
GSE3268	Wachi S	2005	USA	GPL96	5/5
GSE4824	Girard L	2006	USA	GPL96	4/9
GSE11969	Takeuchi T	2008	Japan	GPL7015	35/5
GSE12428	Boelens MC	2008	Netherlands	GPL1708	34/28
GSE12472	Boelens MC	2008	Netherlands	GPL1708	35/28
GSE19188	Philipsen S	2009	Netherlands	GPL570	27/65
GSE19804	Lu T	2010	China (Taiwan)	GPL570	60/60
GSE21933	Chang J	2010	China (Taiwan)	GPL6254	10/21
GSE27489	Kuner R	2011	Germany	GPL570	10/10
GSE29249	Ma L	2011	China	GPL10558	3/3
GSE30219	Rousseaux S	2011	France	GPL570	61/14
GSE31446	Wu H	2011	USA	GPL9244	49/15
GSE31552	Marquardt G	2011	USA	GPL6244	25/25
GSE33479	Mascaux C	2011	France	GPL6480	27/27
GSE33532	Meister M	2011	Germany	GPL570	16/20
GSE40275	Kastner S	2012	Austria	GPL15974	4/43
GSE51855	Takashi Takahashi	2013	Japan	GPL6480	28/4
GSE62113	Tsao M	2014	Canada	GPL14951	7/9
GSE67061	Tong R	2015	China	GPL6480	69/8
GSE74706	Marwitz S	2015	Germany	GPL13497	8/18
GSE103512	Brouwer-Visser J	2017	USA	GPL13158	23/8

**Table 3:** Univariate and multivariate Cox regression analysis for clinical factors in LUSC patients.

Variables	Univariate analysis		Multivariate analysis	
	HR (95% CI)	*P* value	HR (95% CI)	P value
Age (>60/<=60 years)	1.242 (0.871-1.772)	0.232	1.866 (1.205-2.891)	0.005
Gender (male/female)	1.196 (0.868-1.647)	0.275		
Distant metastasis (M1/M0)	3.099 (1.266-7.584)	0.013	2.512 (1.021-6.179)	0.045
Clinical stage (IV/III/II/I)	1.557 (1.131-2.144)	0.007		
Tumor size (T4/T3/T2/T1)	1.651 (1.195-2.281)	0.002	1.516 (1.042-2.205)	0.030
Node metastasis (N3/N2/N1/N0)	1.134 (0.858-1.499)	0.376		
Smoking (heavy/light)	0.656 (0.494-0.872)	0.004		
FOXD1 expression (high/low)	0.750 (0.572-0.983)	0.037	0.706 (0.522-0.956)	0.024

## Data Availability

The raw data can be obtained from public TCGA and GEO database. All data are available from the authors on reasonable request.
